# A realized facilitation cascade mediated by biological soil crusts in a sagebrush steppe community

**DOI:** 10.1038/s41598-023-31967-0

**Published:** 2023-03-23

**Authors:** Wendy M. Ridenour, C. J. Lortie, Ragan M. Callaway

**Affiliations:** 1 Department of Biology, University of Montana Western, Dillon, MT 59725 USA; 2 Department of Biology, York University, Toronto, ON Canada; 3 Division of Biological Sciences, University of Montana, Missoula, MT 59812 USA

**Keywords:** Ecology, Community ecology

## Abstract

Biological soil crusts can have strong effects on vascular plant communities which have been inferred from short-term germination and early establishment responses. However, biocrusts are often assumed to function as an “organizing principle” in communities because their effects can “cascade” to interactions among crust-associated plant species. We conducted surveys and experiments to explore these cascades and found that biocrusts were positively associated with large patches (> 10 m diameter) of a dominant shrub *Artemisia tridentata.* At the smaller scale of individual shrubs and the open matrices between shrubs, biocrusts were negatively associated with *Artemisia*. Juveniles of *Artemisia* were found only in biocrusts in intershrub spaces and never under shrubs or in soil without biocrusts. In two-year field experiments, biocrusts increased the growth of *Festuca* and the photosynthetic rates of *Artemisia*. *Festuca* planted under *Artemisia* were also at least twice as large as those planted in open sites without crusts or where *Artemisia* were removed. Thus, biocrusts can facilitate vascular plants over long time periods and can contribute to a “realized” cascade with nested negative and positive interactions for a range of species, but unusual among documented cascades in that it includes only autotrophs.

## Introduction

Biological soil crusts cover much of the soil surface in the arid and semi-arid regions of the world and have been described an “organizing principle”^1–3^. Soil crusts are highly variable and can be composed of nitrogen-fixing cyanobacteria and cyanolichens, lichens associated with green algae, free-living green algae and diatoms, fungi, mosses, and liverworts^4^. These diverse assemblages are of primary importance to vascular plant communities through their effects on soil water and fertility^1,5,6^. Biological soil crusts (biocrusts hereafter), can increase rainwater infiltration, improve soil moisture retention, increase soil organic matter, and prevent soil erosion^1,3,5^. Mechanistically, as biocrusts hydrate and desiccate, they can release large amounts of carbon and nitrogen into ecosystems^7^. Collectively, these effects can directly facilitate plants.

Biocrusts typically form a complex matrix with vascular plants at both small and large scales^8,9^. This soil/biocrust matrix forms a far more variable “playing field” for plant communities than soil without biocrusts because various plant species respond differently to one physical component or the other^9,10^. The effect of biocrusts corresponds with their composition, and they can have negative, neutral, or positive effects on vascular plants^4,9–14^; see meta-analysis and review by Havrilla et al.^15^. This variation in effects depends on the composition of biocrusts, vascular plant functional groups, and the provenance of the vascular plants. However, in field conditions, many studies have found that the germination and survival of native plant species is higher, or not changed, in patches with biocrusts than in area without crusts^16^. Their facilitative and ecosystem engineering roles have been of substantial interest because of the positive effects of biocrusts on soil water and nitrogen^7,17–19^.

Direct facilitation infers some degree of interdependence among species^20,21^, but simple pairwise direct facilitation can lead to facilitative “interaction chains” that can have complex, but not necessarily obligate, effects through communities. Interaction chains derived from linked direct pairwise interactions between species occur when “…one species directly alters the abundance of a second species, the change in the second species affects a third species, and so on”^22^. These chains can also be characterized as “facilitation cascades” when a series of successive facilitation interactions occur among nested groups of species^23–26^. To our knowledge, all but one of currently reported facilitation cascades have trophic dimensions and have focused on intertidal marine systems (but see Losapio et al.^27^). As an exception, but still trophic in nature, Angelini and Silliman^28^ found that *Quercus virginiana* facilitated epiphytes which in turn dramatically increased invertebrate feeding guilds. Facilitation cascades or positive interaction chains between plants, are likely to occur in semi-arid systems where biocrusts act as “a basal habitat former”^25^, analogous to Altieri et al.^23^ positive effects of cordgrass on mussels, for vascular plant species such as shrubs, which then may have positive effects on other species^29^. Biocrusts can certainly act as an ‘organizing principle’ at multiple scales^1^, and plant species in arid ecosystems can have strong facilitative effects on each other^21^. If these interactions are linked, they can likely also support facilitation cascades among autotrophs. Facilitation cascades, to date, have only focused on links that include consumers or the formation of habitats for animals, but not cascades formed only among autotrophs. Understanding the potential of biocrust-based autotrophic cascades is important^30^ because facilitative cascades can amplify biodiversity^25,26^, and because biocrusts have been extensively destroyed by livestock and recreation^8,31^. Further, the consequences of this damage can be exacerbated by warming temperatures^32^.

We explored possible facilitation cascades in semi-arid shrub steppe in western Montana dominated by the shrub *Artemisia tridentata* Nutt. ssp. *wyomingensis* Beetle & Young (Wyoming big sagebrush) and bunchgrasses, and where biocrusts commonly coexist. As noted, biocrusts can have positive and negative effects, and *A. tridentata* and relevant bunchgrasses have been shown to have facilitative effects on other species^33–38^. Intense competition is also common among these species^35,36,39,40^, and thus interaction cascades may not be entirely facilitative, but instead may be “realized”, which include nested negative and positive interactions^46^.

We integrated two-year field experiments designed to test interactions among biocrusts and two co-dominant vascular species with small-scale and large-scale measurements of spatial patterns of plants, soil water, and soil nitrogen to infer interactions among other species and at a larger spatial scale. We also conducted greenhouse experiments to measure the nitrogen-fixing capacity of the biocrusts. We organized these experiments around the following three predictions. (1) Biocrusts facilitate vascular plants. (2) *Artemisia* facilitates other plants and biocrusts growing under their canopies. (3) Interaction cascades initiated by biocrusts correspond with increased soil nitrogen and moisture. In total, this work identifies direct and indirect effects of biocrusts in the context of change and other key species within the region.

## Methods

### Site

We integrated field experiments and spatial patterns in semi-arid sagebrush steppe at Calf Creek Wildlife Management Area (CCWMA), in western Montana on the east side of the Bitterroot Valley, Montana (46.2805–114.0047; 1380 m elevation). Descriptions of the integrated experiments and spatial pattern measurements are listed in Supplementary Table 1. CCWMA is maintained as winter range for elk (*Cervus elaphus*), and mule deer (*Odocoileus hemionus*). White-tailed deer (*Odocoileus virginianus*) are also common at the site. Cattle are fenced out of CCWMA, but we have observed occasional bovine trespassers. Mean annual precipitation at the site is approximately 30 cm. *Artemisia tridentata* is dominant and occurs in a patchy pattern with areas composed of bunchgrasses and forbs. We focused on the spatial patterns of the native bunchgrasses which were the most common at the site: *Festuca idahoensis* Elmer (Idaho fescue), *Koeleria macrantha* (Ledeb.) Schult. (prairie junegrass), *Pseudoroegneria spicata* (Pursh) Á. Löve (bluebunch wheatgrass), *Hesperostipa comata* (Trin. & Rupr.) (needle-and-thread grass), *Leymus cinereus* (Scribn. & Merr.) (Great Basin wild rye), and *Poa secunda* (J. Pres) (Sandberg’s bluegrass). We then focused on *F. idahoensis* (hereafter *Festuca*) in experiments testing the effects of *Artemisia* and biocrusts. The large-scale patches of both *Artemisia* and herbaceous communities were typically 10 to > 100 m in cross section when visually surveyed during experimental setup. We also observed relatively low abundances of the exotic invaders *Centaurea stoebe* and *Bromus tectorum*.

### Spatial patterns

Based on other studies finding that biocrust cover increased with shrub cover at large scales, but decreased with shrub cover at small scales^43^, we measured spatial patterns at the following two scales: at the large scale of patches containing many *Artemisia* shrubs vs. similar sized patches without any shrubs, and at the small scale of individual *Artemisia* understories vs. small open areas within *Artemisia* patches. Because our observations suggested that *Artemisia* might create interstitial spaces with low grass density, we sampled at the large scale to determine whether bunchgrass species or biocrusts were associated or disassociated with *Artemisia* patches. Because shrub canopies might shade biocrusts, we sampled at the small scale to determine whether bunchgrass species or biocrusts within patches were associated or disassociated with *Artemisia* individuals. At the large scale, 30 1 × 1 m quadrats were randomly located within haphazardly chosen *Artemisia* and grassland patches. In each quadrat, the number of each of the bunchgrass species listed above were counted and the percent cover of *Artemisia* and biocrust was estimated visually to the nearest 10%. We used the “basic distance” approach^41^ with 20 m transects run through *Artemisia* patches to randomly choose shrubs for spatial associations and sampled vegetation at random points chosen within each of the 5 m section blocks on each transect. We located a 30 × 30 cm quadrat under the north quadrant of each of 50 *Artemisia* shrubs, and a 30 × 30 quadrat in the nearest open space to the north of each of these sampled *Artemisia* individuals. In each quadrat, we visually estimated the percent cover of biocrust to the nearest 5% and counted all individuals of the grass species listed above. The species were separated into those with or without flower stalks. Finally, to estimate the effect of crust on juvenile *Artemisia*, we sampled five 20 × 100 m areas for juveniles (< 20 cm tall) and recorded whether they were in the open, under adult shrubs, or within a biocrust patch.

### Field experiments

To experimentally test the effects of biocrusts on *Artemisia*, we established 20 30 × 30 cm plots in areas of undisturbed biocrust cover in June 1996. In half of these plots, the biocrusts were removed and in the other half biocrusts were left intact. A one-year-old *Artemisia* plant was transplanted into the center of each plot. These juvenile *Artemisia* had been grown from locally collected seed in a blend of silica sand and soils collected from the field study site in a greenhouse before transplanting. Each plot was caged with 12-gauge wire mesh to prevent trampling and herbivory by native ungulates. These transplants were left in place for two years, but after a year, our observations suggested that growth was very slow, perhaps too slow to provide detectable comparisons of biomass. Therefore, we measured photosynthetic rates of *Artemisia* towards the end of the growing season (mid-September) in 1996 and again in 1997 using a LI-COR 6200 portable infrared gas analysis system. We placed entire *Artemisia* seedling shoots into a custom-made 1.2 L cuvette and exposed them to full sunlight without cloud interference. After two years, the aboveground biomass of *Artemisia* transplants was harvested, dried at 60 °C, and weighed.

To experimentally test the effects of biocrusts on *Festuca*, we established 20 30 × 30 cm plots in areas of undisturbed biocrust cover in June 1996. In half of these plots, the biocrusts were removed and in the other half they were left intact. A one-year-old *Festuca* plant was transplanted into the center of each plot. These had been grown from locally collected seed in a greenhouse in a blend of silica sand and soils collected from the field study site before transplanting. Each plot was caged with a 12-gauge wire mesh to prevent trampling and herbivory by native ungulates. These transplants were in situ for two years.

To test the effect of *Artemisia* on *Festuca*, we randomly selected 60 mature *Artemisia* shrubs and 30 were removed and 30 left undisturbed. We also selected 60 open areas with biological soil crust, at least two meters away from the nearest *Artemisia* for shade treatments. Half of these 60 open areas were covered with a shade screen treatment using shade cloth that blocked approximately 30% of sunlight and half were left without shade screens. In each of these 120 plots, a single one-year old *Festuca* plant was transplanted. In mid-September 1997, we measured *Festuca* survivorship, the proportion of grazed plants, and harvested and weighed aboveground biomass. Any *Festuca* that showed clear signs of leaf herbivory was counted as grazed.

We measured soil moisture gravimetrically across all treatments in both the *Artemisia*-effect and soil crust-effect experiments. Soil samples were collected in all plots in mid-September 1997 when there had been no precipitation for over a week, from a depth of 5–10 cm. Each sample was weighed, dried at 100 °C for 2 days and weighed again for gravimetric soil moisture. In mid-October 2022, again roughly a week after light precipitation, we sampled soil from 5 to 10 cm depth beneath *Artemisia*, beneath biocrusts, and in the open. Samples were weighed, dried at 100 °C for 2 days and weighed again for gravimetric soil moisture.

### Greenhouse experiments

We conducted a greenhouse experiment to test the effects of biocrusts on the germination and establishment of *Festuca*. The greenhouse was on the University of Montana campus (46.8609, − 113.98401). In June 1996, soil was collected from the field site and was added to 50 × 25 × 10 cm rectangular trays. Biocrusts removed for the field experiment were established on half of these trays, and the other half were left without biocrusts. Fifty *Festuca* seeds were sown in each tray, spray-misted twice per day until seeds germinated, and were subsequently watered two times per week for the duration of the experiment. Germination was monitored, and after 120 days, all surviving *Festuca* plants were counted again as “established”, then harvested, dried at 60 °C, and weighed.

### Laboratory experiments

We conducted laboratory experiments to determine the cyanobacterial composition of biocrusts and to estimate the nitrogen-fixing capacity of biocrusts over a range of temperatures and water saturation. Biocrusts were collected from the field site. Belnap^8^ stated that “cyanobacteria form the matrix of biological soil crusts”, thus we cultured and isolated cyanobacterial species on 1% agar plus medium D and the dominant genera were identified using phase contrast microscopy.

For nitrogen-fixation, biocrusts were maintained in enclosed growth chambers under controlled temperature and light similar to those typical in the field. The source of photosynthetically active radiation (PAR) in the 400–700 nm range was from very high output cool-white florescent lamps. Sylvania black light fluorescent lamps (peak output 365 nm, range 280–360 nm) provided a source of UVA radiation, and Westinghouse FS—40 fluorescent lamps (peak output 310 nm, range 280–360 nm) were the source of UVB radiation. Biocrust samples used for the acetylene reduction assays were 2 × 2 cm. The cuvettes used for crusts were 12 ml, injected with 1 ml acetylene (C_2_H_2_), and incubated for 1 h. Following incubation, gas samples were withdrawn and C_2_H was quantified using a Gow-Mac 69–750 gas chromatograph. Biocrust nitrogenase activity was measured over a range of temperatures from 0 to 45 °C, and a range of biocrust water saturation from about 10% to full saturation.

### Statistical analyses

All statistics were performed in SigmaPlot 14.5 (Systat Software, Inc. 2020) and cross-validated in R 4.2.1^42^. Linear regressions were used to test the relationships between vascular plant cover in large-scale (> 10 m diameter) patches for both herbaceous plants and *Artemisia*. For the relationship between *Artemisia* juveniles and biocrust, we used a Chi-square test, and for comparisons of herbivory on *Festuca* under *Artemisia* and in the open we used a Fischer’s exact test. For all other pairwise comparisons, we used t-tests when data were normally distributed and Mann–Whitney Rank Sum tests when assumptions of normality were not met—these are noted in the results. For the four concomitant treatments in the field experiment testing the effects of *Artemisia* on *Festuca*, the data failed the Shapiro–Wilk normality test, so we used a Kruskal–Wallis one way ANOVA on ranks followed by Dunn’s Method pairwise multiple comparisons. Our goal for N-fixation was simply to determine the capacity of our biocrusts to provide nitrogen to the system, therefore we grouped rates by two ranges of temperatures and two ranges of water saturation and presented these rates in box plots. For comparisons of soil water among *Artemisia*, biocrusts and soil without biocrusts in 2022, data were not normally distributed so we used a Kruskal–Wallis one way ANOVA on ranks followed by Tukey tests for pairwise comparisons. For comparisons of ammonium and nitrate in soils we used one way ANOVA followed by Holm-Sidak pairwise multiple comparisons.

## Results

### Spatial patterns

**Figure 1:**
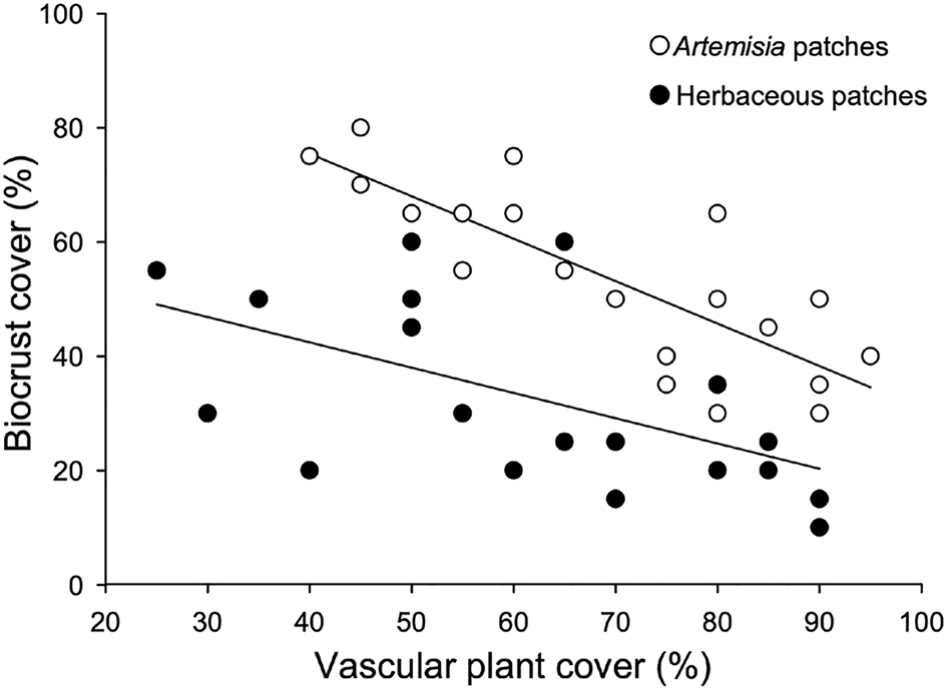
Regressions between vascular plant cover and biocrust cover at large scales, with open symbols showing the relationship between *Artemisia tridentata* shrub cover and biocrust cover (R^2^ = 0.66; n = 20; P < 0.001) and closed symbols showing the relationship between herbaceous cover and biocrust cover (R^2^ = 0.32; n = 20; P < 0.001).

Biocrust cover was 54 ± 4% in large patches of *Artemisia* vs. 34 ± 4% in large open patches without *Artemisia* (Mann–Whitney U = 68.5; P > 0.001; Fig. 1). In each patch type, increasing vascular plant cover corresponded with decreasing biocrust cover (Fig. 1). At this large scale,the six selected bunchgrass species were twice as common in open patches without *Artemisia* as in *Artemisia* patches, 19.1 ± 1.4 vs. 10.4 ± 1.9 individuals per m^2^ (t = − 5.199, P < 0.001). *Festuca*, the target experimental species, occurred at similar densities in both patch types; 3.0 ± 0.9 in herbaceous patches vs. 1.6 ± 0.4 in *Artemisia* patches (Mann–Whitney U = 192.0; P = 0.836).

In contrast to the large-scale patterns, at the scale of individual *Artemisia* shrubs, biocrusts were less abundant under *Artemisia* than in the open matrix surrounding *Artemisia*. Within *Artemisia* patches, and under individual *Artemisia* shrubs, biocrust cover was 39 ± 4% vs. 84 ± 3% in the inter-shrub open matrix (Mann–Whitney U = 204.0, P < 0.001). The richness of the six selected bunchgrass species under *Artemisia* was 0.9 ± 0.2 vs. 1.9 ± 0.1 per 0.25 m^2^ in the open matrix (Mann–Whitney U = 602.50, P < 0.001). The total density of these species was 1.9 ± 0.3 per m^2^ under *Artemisia* vs. 4.7 ± 0.4 in the open matrix (Mann–Whitney U = 531.0, P < 0.001). There were more reproductive *Festuca* in the open matrix than under *Artemisia* (0.5 ± 0.1 vs. 0.3 ± 0.1 per m^2^; Mann–Whitney U = 1019.000; P = 0.050), but non-reproductive *Festuca* were four times more abundant under *Artemisia* (0.8 ± 0.2 vs. 0.2 ± 0.1 per m^2^; Mann–Whitney U = 947.0; P = 0.004).

We recorded 23 juvenile *Artemisia* in *Artemisia* patches, none of which were under mature *Artemisia* or in open soils without biocrust, and all of which were in biocrusts. Based on this observed number and the expected numbers derived from the 84% biocrust cover in the interstitial spaces among *Artemisia* shrubs, biocrusts correlated positively with the recruitment of *Artemisia* (Chi-square test, χ^2^ = 4.41, P = 0.036).

### Field experiments

**Figure 2:**
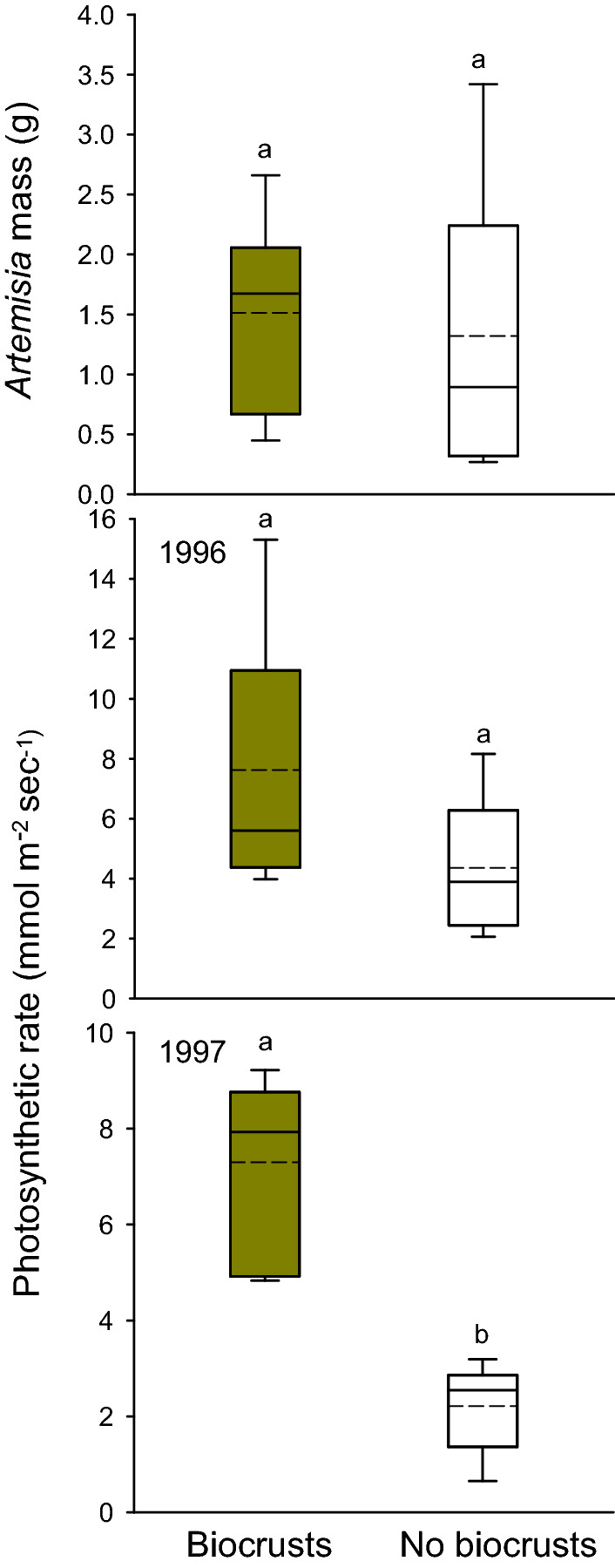
Aboveground biomass of *Artemisia tridentata* juveniles grown for two years in locations with biocrusts intact and biocrusts removed, and photosynthetic rates of one-year-old and two-year-old *Artemisia* in the same treatments. Boxplots represent the interquartile range and median, and points represent outliers, and the dashed lines indicate means. Shared letters indicate no statistical difference between treatments and dashed lines show the means.

The aboveground biomass of juvenile *Artemisia* planted in biocrusts did not differ from that of *Artemisia* planted where biocrusts had been removed (Fig. 2a; t = 0.386; df = 2,14; P = 0.706). However, these same *Artemisia* planted in biocrusts photosynthesized at higher rates in 1997 than those planted where biocrusts had been removed (Fig. 2c; t = 6.301; df = 2,11; P < 0.001), but this was not the case in 1996 (Fig. 2b).

**Figure 3:**
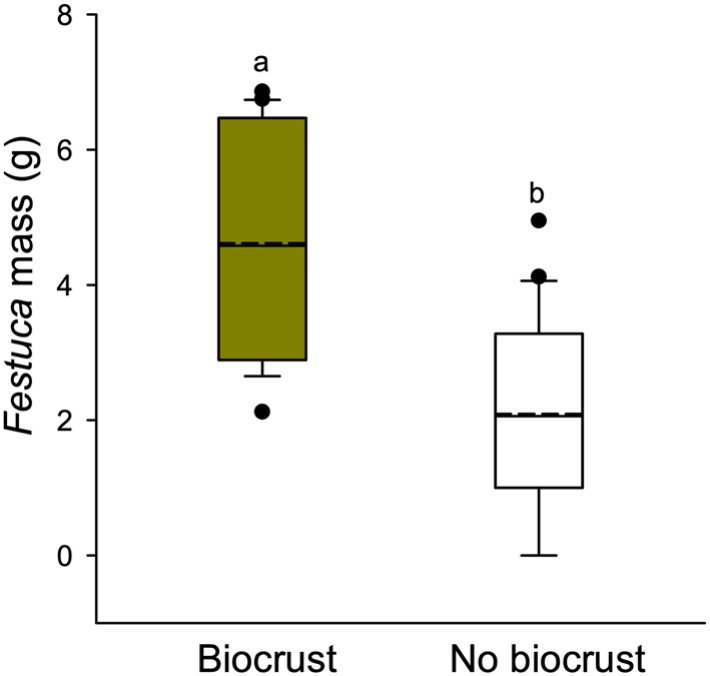
Aboveground biomass of *Festuca idahoensis* planted in intact biocrusts and where biocrusts had been removed. Boxplots represent the interquartile range and median, and points represent outliers, and the dashed lines indicate means. Shared letters indicate no statistically significant difference between treatments.

*Festuca* planted in biological soil crust was almost twice as large after two growing seasons as those growing in no biological soil crust treatments (Mann–Whitney U = 55.0; df = 2, 39; P < 0.001; Fig. 3).

**Figure 4:**
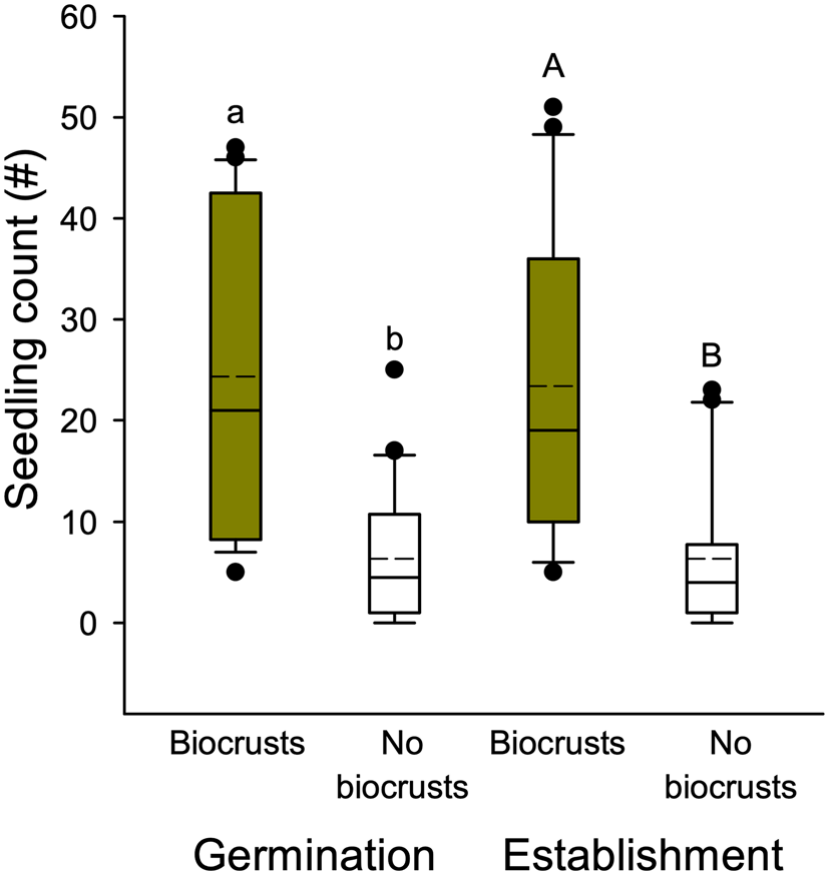
Emergence and recruitment of *Festuca idahoensis* on greenhouse experiments in biocrusts from the field placed on soils and in soils without biocrusts. Boxplots represent the interquartile range and median, and points represent outliers, and the dashed lines indicate means. Shared letters indicate no statistical difference between treatments and the dashed lines shows the means.

*Artemisia* facilitated *Festuca* after two growing seasons, as *Festuca* biomass under *Artemisia* canopies was roughly twice as much as those in the open (Kruskal–Wallis ANOVA on Ranks, Dunn’s pairwise, Q = 2.815, P = 0.029; Fig. 4). *Festuca* biomass under *Artemisia* canopies was also different than that of *Festuca* planted under shade placed in the open (Q = 3.494, P = 0.003). However, the biomass of *Festuca* planted under *Artemisia* canopies was not different than those planted where *Artemisia* had been removed (Q = 1.960, P = 0.300).

### Greenhouse experiment

**Figure 5:**
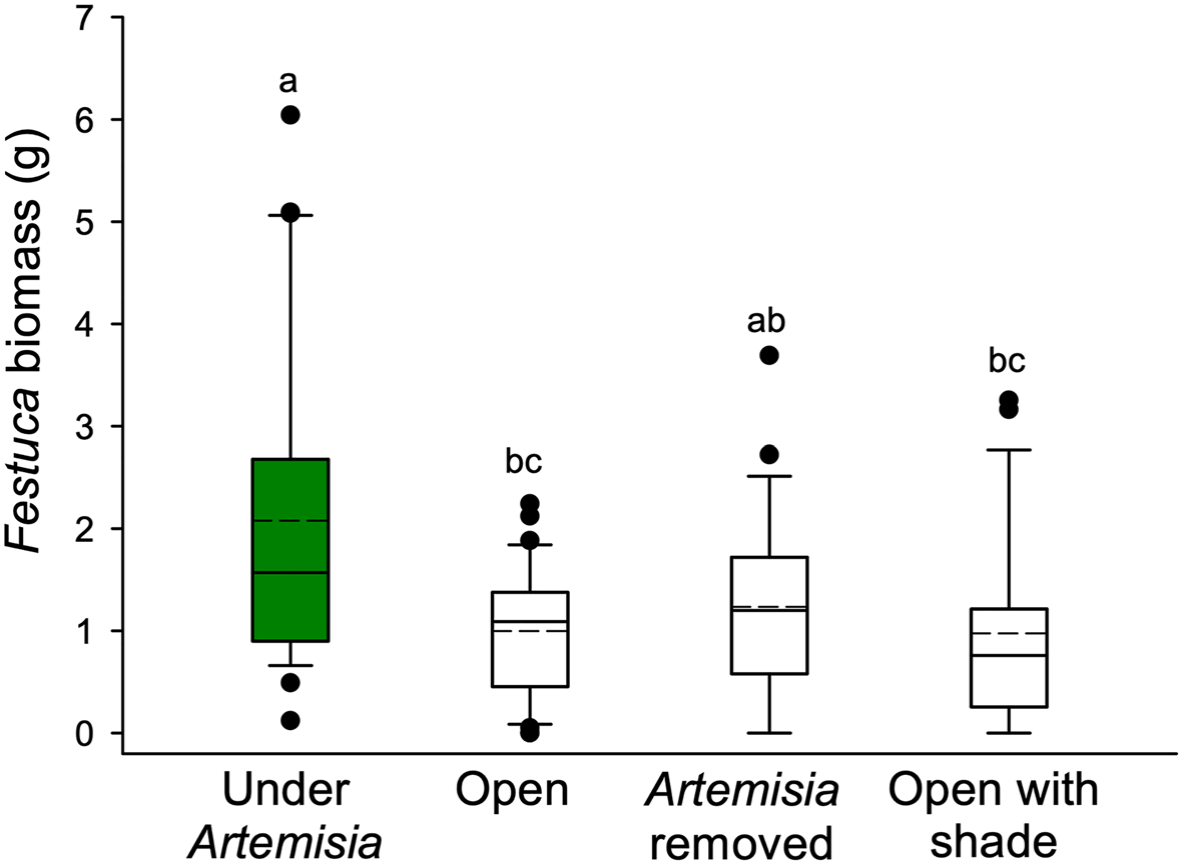
Aboveground biomass of *Festuca idahoensis* experimentally planted for two years under *Artemisia tridentata*, in the open matrix around *Artemisia* (without biocrusts), where *Artemisia* was experimentally removed, and in the open (without biocrusts) with 30% artificial shade provided. Boxplots represent the interquartile range and median, and points represent outliers, and the dashed lines indicate means. Shared letters indicate no statistical difference among treatments and dashed lines show the means.

Biocrusts strongly facilitated *Festuca* in the greenhouse experiment. When grown in crusts, *Festuca* germination was roughly five times higher than without crusts (Fig. 5; Mann–Whitney U = 55.000*,* P < 0.001), and establishment was almost four times greater in the biocrust treatment (t = 4.538; df = 2,38; P < 0.001).

### Potential mechanisms

#### Nitrogen

**Figure 6:**
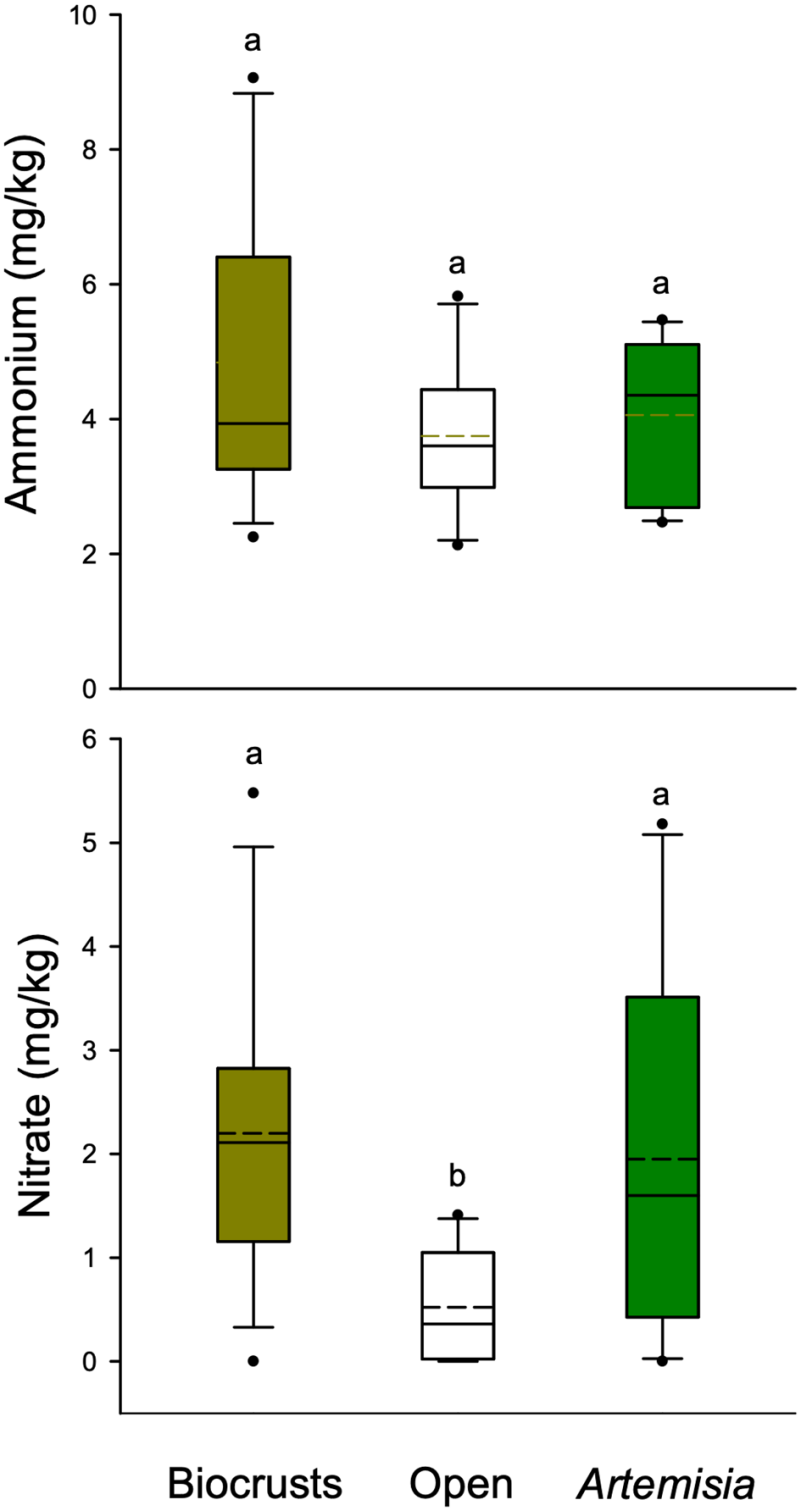
Soil concentrations of ammonium and nitrate 5–10 cm beneath *Artemisia*, beneath biocrusts in the open, and in the open without biocrusts. Boxplots represent the interquartile range and median, and points represent outliers, and the dashed lines indicate means. Shared letters indicate no statistical difference among treatments and dashed lines show the means.

Laboratory cultures of biological soil crust cyanobacterial species found four predominant genera of cyanobacteria in the biocrusts. Two of these genera, *Scytonema* and *Nostoc*, are strong nitrogen fixers, and *Microcoleus* and *Phormidium* are weak nitrogen fixers. Laboratory assays of biocrust nitrogen fixation suggest that under a broad range of natural field temperature and moisture conditions, sagebrush steppe biocrusts fix a potentially significant amount of nitrogen (Fig. S1). In 2022, concentrations of ammonium did not differ among soils under biocrusts, under *Artemisia* or in the open (Fig. 6; one way ANOVA, F = 1.365; df = 1,29; P = 0.271). Nitrate concentrations were higher under biocrusts (t = 2.917; P = 0.020) and *Artemisia* (t = 2.376; P = 0.048) than in the open (F = 4.754; df = 1,29; P = 0.016). There was no difference in nitrate in soils beneath biocrusts and *Artemisia* (t = 0.435; P = 0.667).

#### Water

In 1997, when soils were much wetter overall, biocrusts had no effect on soil moisture content in mid-September 1997, with the mean percent water content of soil under biocrusts at 11.8 ± 1.1% and soil without biocrusts at 11.7 ± 1.5% (t = 0.0408; df = 26.1, P = 0.968). In 2022, when soils were much drier overall, soil moisture was 4.0 ± 0.7% under biocrusts vs. 2.3 ± 1.3% in the open without biocrusts (Kruskal–Wallis one way ANOVA on Ranks, H = 11.821, df = 2, P = 0.003; Tukey test = 0.003). In 1997, soil moisture under *Artemisia* did not differ from that in the open (11.5 ± 0.5 vs. 11.0 ± 0.4; t = 0.713, df = 3,61, P = 0.479). In 2022, soil moisture was 2.4 ± 0.2% under *Artemisia*, which did not differ from that in the open without biocrusts (Tukey test, P = 0.712), but was lower than that under biocrusts (Tukey test, P = 0.033).

#### Herbivory

Of the surviving *Festuca* planted under *Artemisia*, 32% (9 of 28) were damaged by herbivores, vs. 83% (19 of 23) in the open matrix (Fischer’s exact test = 0.002; P < 0.001).

## Discussion

**Figure 7:**
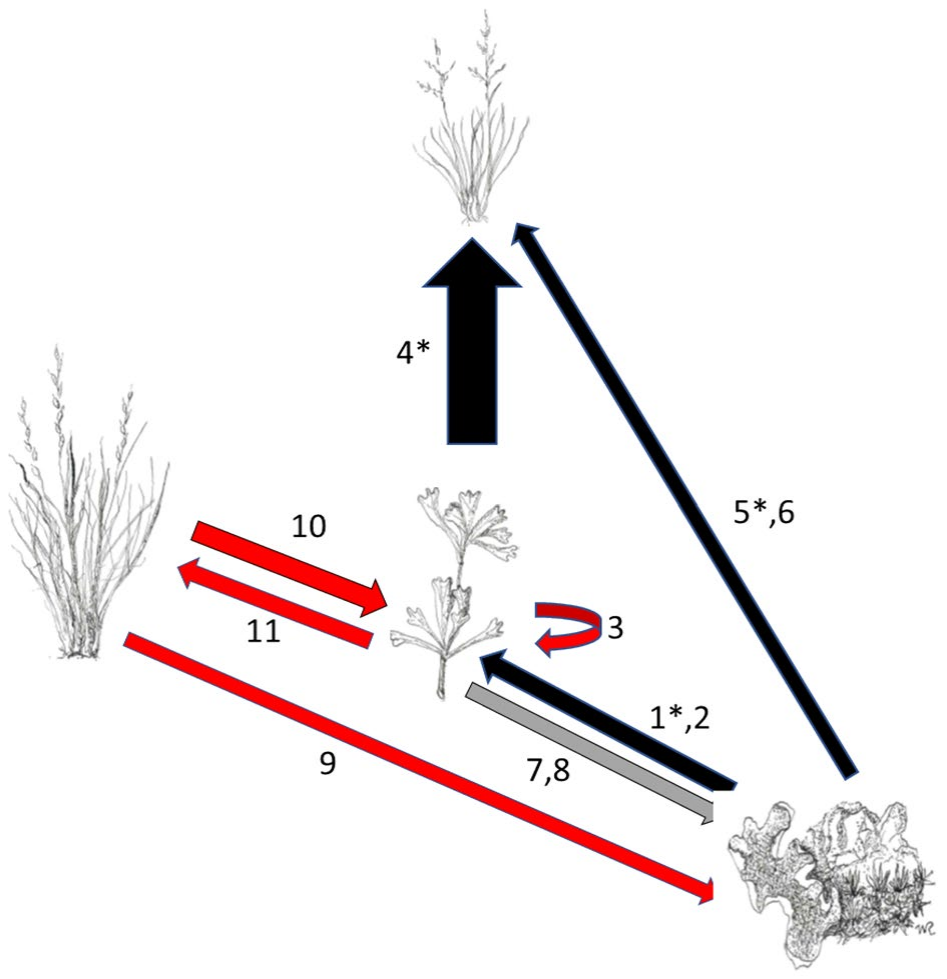
Flow diagram for interactions among biocrusts, *Artemisia tridentata* (center drawing), *Festuca idahoensis* (top drawing), and “other” grass species. Arrow width roughly represents the intensity of the interaction, black arrows indicate positive effects, red arrows represent negative effects, and the gray arrow represents a scale-dependent effect. Numbers designated the specific study component providing evidence, with * representing experimental evidence. 1* = biocrust removal experiment, 2 = strong spatial association of *Artemisia* seedlings with biocrust, 3 = spatial disassociation of *Artemisia* seedlings with adult *Artemisia*, 4* = *Artemisia* removal experiment, 5* = biocrust removal experiment, 6 = greenhouse experiment with and without biocrust, 7 = small-scale spatial disassociation of biocrusts with *Artemisia*, 8 = large-association of biocrusts with *Artemisia*, 9 = spatial disassociation of biocrusts with bunchgrass species as a group at large-scales, 10 = spatial disassociation between *Artemisia* and bunchgrass species as a group, 11 = spatial disassociation between *Artemisia* and bunchgrasses at large scales.

The most salient aspect of our results is that biocrusts facilitated a key vascular plant in this system, the shrub *Artemisia tridentata.* Through this facilitation, biocrusts appeared to amplify a cascade of extended effects ranging from facilitative effects on *Festuca idahoensis* to apparent competitive effects on other bunchgrasses, juvenile *Artemisia*, and biocrusts themselves; the latter three all based on spatial patterns (Fig. 7). Importantly, the effect of *Artemisia* on biocrusts may depend on the scale at which patterns are measured. At larger scales, the negative relationship between *Artemisia* shrubs and grasses in general appeared to create interstitial spaces around shrubs that actually increased the abundance of biocrusts in large shrubby patches relative to large herbaceous patches, despite the negative effects of individual shrubs within large patches. This may be because bunchgrass density was roughly four times less in these interstitial spaces than in the large open patches without *Artemisia*. Soliveres and Eldridge^43^ described similar scale-based differences in positive and negative spatial associations among shrubs and biocrusts in Australia, with biocrust cover and diversity increasing with shrub cover at large scales, and negative effects at small scales.

In addition to indirect facilitation through *Artemisia*, biocrusts also strongly directly facilitated *Festuca*, evidenced by higher germination and establishment in the greenhouse, and higher growth in the field experiment. Secondary components of cascades included facilitative effects of *Artemisia* on *Festuca*—increased growth of *Festuca* under *Artemisia* in field experiments and spatial associations between non-flowering *Festuca* and *Artemisia* shrubs in the field. Other cascading outcomes, based on spatial associations, included the apparent suppression of other bunchgrasses and juvenile *Artemisia* by adult *Artemisia*, with all suggesting that biocrusts may drive important cascades in intermountain shrub steppe.

Components of these chains of interactions are consistent with facilitation cascades described in shallow intertidal zones and sea beds^23,44^. The latter described a scenario in which cordgrass establishes and then facilitates a number of other species including mussels, snails, and seaweeds. Much like *Artemisia* in our case, facilitated mussels increase the densities of other species such as amphipods and barnacles (also see Altieri et al.^24^). They suggested that cordgrass acted as a “primary facilitator”, perhaps like biocrusts, and mussels acted as a “secondary facilitator”, perhaps like *Artemisia*. An important difference in our system, however, was that biocrust-facilitated *Artemisia* appeared to have many negative, or competitive, effects on most bunchgrass species. Thus, our results might be more like results for “multiple, independent cascades” in mangrove-dominated systems that appeared to be initiated by “a single basal facilitator”^45^. The spatial patterns of the large suite of species we measured also suggest a cascade more in line with that of Gribben et al.^46^. They presented evidence for “a realized facilitation cascade” as a “function of nested negative and positive interactions” that vary as the density of key species varies, rather than a “collection of hierarchical positive interactions”. Although we do not explore this, these complex negative and positive interactions have the potential to maintain community diversity through intransitive interactions^47^.

Other facilitation cascades in the literature generally have more obligate relationships than the biocrust → *Artemisia* → *Festuca* cascade explored here. In part, this may be because none of our cascade components are consumers that can obviously have strong, obligate relationships with prey abundances. Each of our autotrophic components can certainly exist without the others, thus, the cascades suggested by our results are relatively more facultative and contextual than others reported in the literature.

The variable negative and positive interactions in a realized facilitation cascade^46^ might be related to facilitation and competition being highly context dependent, and such context dependence appears to be very much the case for the effects of biocrusts in general^15^. In a large-scale multi-factorial field experiment in intermountain prairie, vegetation very similar to ours, biocrusts inhibited the overall emergence of four different exotic seedlings in a wet year, but not a dry year, and had no effect on the emergence of four different native species^9^. The effects of biocrusts in rosemary scrub in Florida vary among species, distance from shrubs, and time since fire^48^. Experimental removal of biocrusts from around established *Bouteloua gracilis* bunchgrasses reduced their performance^49^. These kinds of variable effects are highlighted in a meta-analytical review which found that biocrusts dominated by lichens, such as ours, inhibited overall plant performance—on average^15^. In this review, biocrusts had more positive effects on plant species without mutualistic nitrogen-fixing bacteria, such as our *Festuca* and *Artemisia* than species with these symbionts. More importantly, they found that lichen-dominated biocrusts across studies suppressed germination, the opposite of what we found in the greenhouse experiment for germination and establishment, but improved growth, as we found in the field for *Festuca*, and corresponded with the higher photosynthetic rates of *Artemisia* in biocrusts in the field.

The effects of *Artemisia* on native bunchgrasses are less studied than those of biocrusts, but they are also variable. Early studies indicated that *Artemisia* effects are generally, but conditionally, competitive^39,50,51^, and in a detailed experimental study the roots of *Artemisia* outcompeted those of *P. spicata* for soil nitrogen^40^. However, others have found that *Poa secunda*, a species apparently excluded from *Artemisia* understories in our study, was more common under *Artemisia* than in the open, but only at the driest sites^52^. In this study, *Elymus elymoides* abundance was higher under *Artemisia* canopies than in open interspaces at both drier and wetter sites. In experimental manipulations, Poulos et al.^53^ found that *Artemisia* facilitated the native forb, *Penstemon palmeri*, but this generated complex interactions with other herbaceous species in the understory, another aspect of interaction cascades that we did not pursue. In eastern Oregon, a wide range of different functional groups of vascular plants are positively associated with *Artemisia*^37^ much as found for many other shrub species^54,55^. Furthermore, there is evidence that *Artemisia* can have positive allelopathic (i.e., biochemically mediated) effects on conspecifics^56^ and negative allelopathic effects on understory grasses^57^. As a group, our six selected native bunchgrass species were negatively associated with *Artemisia*. All of this variability in shrub effects considered the facilitative effect of biocrusts on *Artemisia* clearly has the potential to affect a diverse variety of interaction cascades in addition to ours. In fact, although we focused solely upon autotrophs in our study, because less is known about cascades among autotrophs, *Artemisia* is a keystone species for consumers. *Artemisia* provides habitat for obligate consumers including the greater sage-grouse (*Centrocerus urophasianus*), sagebrush sparrow (*Artemisiospiza nevadensis*), sage thrasher (*Oreoscoptes montanus*), and pronghorn antelope (*Antilocapra americana*)^58^. As such, the maintenance of larger scale community structure and diversity in sagebrush steppe may be affected by even more extended and trophic biocrust-*Artemisia* facilitation cascades.

Our results indicating a positive effect of biocrusts on *Artemisia* are, to our knowledge, somewhat atypical in the literature; however, a broad survey across the Great Basin found that biological crusts correlated positively with greater growth of sagebrush canopies^59^. Zhang and Belnap^60^ found that biocrusts improved the concentration of various nutrients in the leaf tissue of two Asian shrub species. And in a meta-analysis, averaged across all biocrust types, biocrusts facilitated the growth of non-nitrogen-fixing shrubs, such as *Artemisia*^15^.

*Artemisia tridentata* with biocrusts cover vast parts of western North America, and yet we know surprisingly little about the effects of this shrub species on biocrusts. Condon et al.^61^ found that *Artemisia*-dominated plant communities were associated with “rapidly reproducing biocrusts”. Navas Romeroa et al.^62^ found positive spatial associations among different shrub species and biocrusts at some sites, but neutral associations at other sites. Others have reported higher biocrust development under *Artemisia ordosica* canopies in China^63–65^. In New South Wales, Australia increasing shrub cover, at the large scale of “sites”, corresponded with increasing biocrust richness and cover, much like the large-scale patterns we report here^43^. However, much as we found at small scales, biocrusts responded negatively to shrubs as compared to open patches^43^.

In our study, biocrust cover decreased as vascular plant cover increased in both large-scale *Artemisia* and large-scale herbaceous patches, consistent with the small-scale negative relationship between *Artemisia* individuals and biocrust cover. She et al.^66^ found that biocrust cover rapidly increased with the cover of shrubs, dominated by *A. ordosica* when shrub productivity was low. As shrub productivity increased, the cover of biocrust did not decrease, but stabilized. In contrast, and like our results, biocrusts were abundant at low herbaceous productivity but declined rapidly with increasing herbaceous productivity. This corresponded with a strong decline in light associated with increasing herbaceous productivity, but not with increasing shrub productivity. One overlooked potential component of our cascade is that *Artemisia* may have substantially altered the composition of biocrusts, which might have had consequences for vascular plants, but which we did not measure. In the field, we observed that biocrusts under mature *Artemisia* canopies appeared to contain much more moss, *Tortula roralis*, than biocrusts in the open. Moss dominated crusts might have weaker positive or stronger competitive effects on vascular plants than cyanobacteria and lichen-dominated biocrusts, which were more common in the open, and with which we conducted laboratory measurements.

Biocrusts can improve the retention of soil water and increase concentrations of soil nitrogen^1,6,19^. Our measurements were only taken as snapshots in time but suggest that both of these processes may have been important. When soils were wet, in 1997, there were no differences in percent moisture among *Artemisia*, biocrusts, and open soil with no biocrusts. But, in 2022, when soils were much drier, soil under biocrusts was wetter than either in the open or under *Artemisia*, suggesting that biocrusts may help retain water. Also in 2022, soil nitrate concentrations were almost three times higher under biocrust as in the open, but nitrate under *Artemisia* was as high as under biocrusts. Interestingly, the mechanism by which *Artemisia* facilitated *Festuca* also appeared to be substrate related, perhaps via nitrogen as suggested by our results; i.e., in treatments where *Artemisia* was removed the biomass of *Festuca* was not significantly different than where it was not removed, perhaps due to its legacy effects on soils. Furthermore, when shade was provided to open sites where shrubs had not affected soils, *Festuca* biomass did not increase. However, shrubs can affect other species via many other effects on substrate that we did not measure^67^. Furthermore, because the species involved have different lifespans and recruitment requirements, these effects must have temporal components which are best considered in the context of priority effects^68^ but exploring priority effects are beyond the scope of this study.

For simplicity, we treated biocrusts as a single entity, but biocrusts are exceedingly complex communities themselves, being dynamic combinations of nitrogen-fixing cyanobacteria and cyanolichens, other bacteria, green algae, fungi, mosses, and liverworts^5^. Pietrasiak et al.^5^ compared functions among 10 different categories of biocrusts, each which certainly can vary in composition, and found that cyanolichen-dominated crusts fixed nitrogen at higher rates than all other crust types, photosynthesized at higher rates than most other crust types, and generated soil aggregate stability as high as any other crust type. The biocrusts in the open at our sites were dominated by lichens and combined with our identification of cyanobacteria indicate that we predominantly measured cyanolichen crusts. Importantly, different biocrusts, or different compositions of cyanolichen crusts, could potentially generate different and unique interaction chains and cascades, and this is an opportunity for deeper context specific research in arid and semi-arid systems that support biocrusts.

## Supplementary Information

Supplementary Information.

## Data Availability

Data are published at https://knb.ecoinformatics.org/view/doi%3A10.5063%2FF11J986P#urn%3Auuid%3A1860bf08-dc31-438f-8f68-91b6649bb0aa.
