# Supplementary material for: A realized facilitation cascade mediated by biological soil crusts in a sagebrush steppe community
Source: Sci Rep. 2023 Mar 23;13:4803. doi: 10.1038/s41598-023-31967-0 (PMC10036522; doi:10.1038/s41598-023-31967-0)
Supplement: Supplementary file 1 — Supplementary Information. [file 41598_2023_31967_MOESM1_ESM.docx]

**Supplementary Information**

Supplementary Table 1.

**Study component**

**Experiments**

1. Effect of biocrusts on *Artemisia tridentata*.
2. Measured *Artemisia* biomass.
3. Measured *Artemisia* photosynthetic rates.
4. Effect of biocrusts on *Festuca idahoensis*.
5. Measured Festuca biomass.
6. Measured whether *Festuca* experienced herbivory.

**Spatial Associations**

1. Large-scale patterns.
2. Measured association between bunchgrass patches and biocrusts.
3. Measured association between *Artemisia* patches and bunchgrasses.
4. Measured association between *Artemisia* patches and biocrusts.
5. Small-scale patterns.
6. Measured association between individual *Artemisia* shrubs and bunchgrasses.
7. Measured association between individual *Artemisia* shrubs and biocrusts.


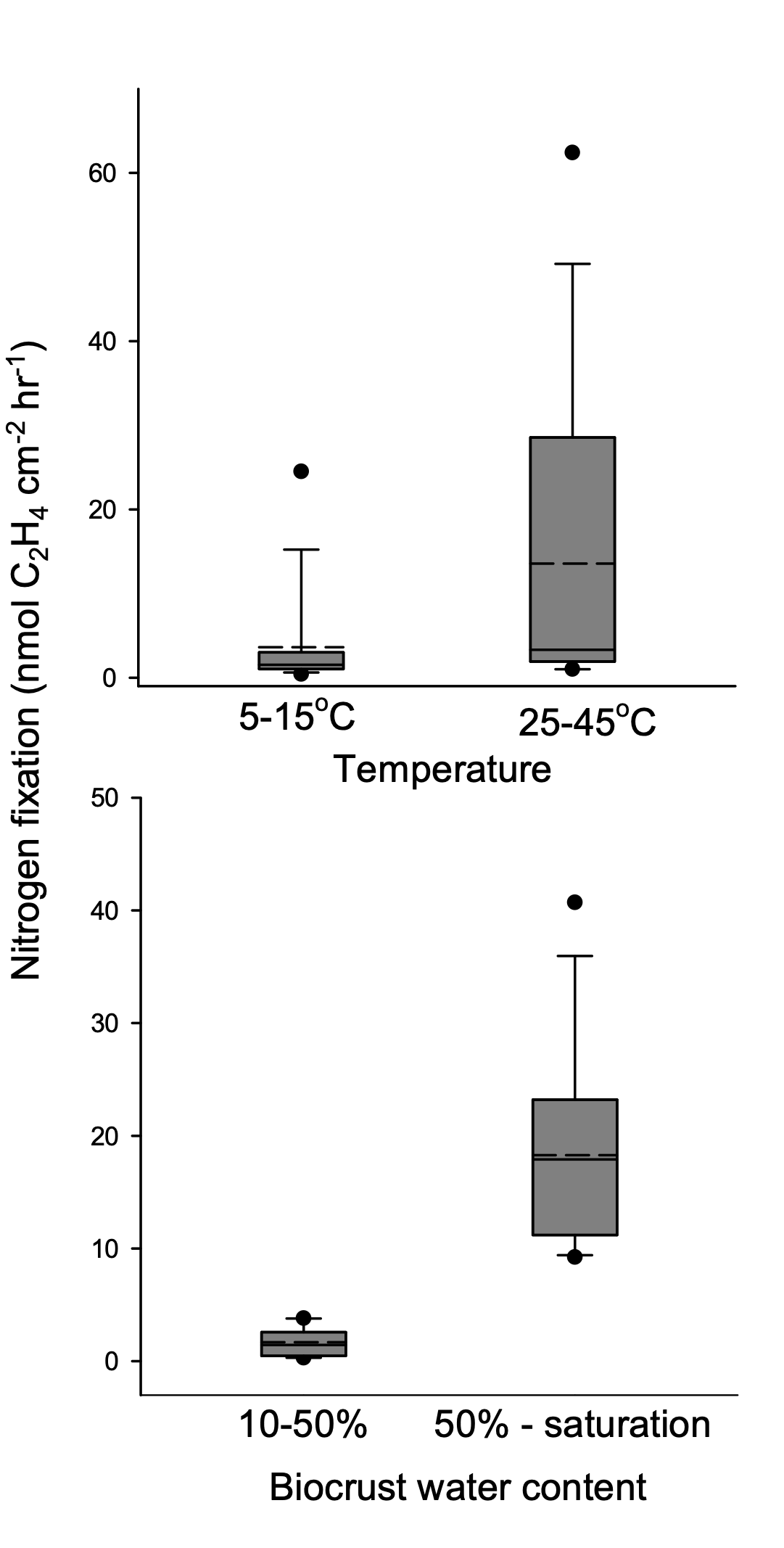


**Figure S1**. Biocrust N-fixation measured as acetylene reduction for biocrusts in controlled laboratory conditions measured between 5 -15°C and 25 - 45°C at 25% water saturation, and at 10-50% and 50-100% water saturation at 25°C. Boxplots represent the interquartile range and median, and points represent outliers, and the dashed lines indicate means.
